# Gray-zone late gadolinium enhancement greatly enriches the prediction of ventricular arrhythmia; a cardiovascular MRI study

**DOI:** 10.1186/1532-429X-14-S1-O17

**Published:** 2012-02-01

**Authors:** Asghar Fakhri, Harish Manyam, Mohammad A Rana, Sourabh Prabhakar, Ronald B Williams, William Belden, John Chenarides, Kenneth Judson, Christopher Bonnet, Robert W Biederman

**Affiliations:** 1 Allegheny General Hospital, Pittsburgh, USA

## Summary

Gray-zone imaging for VT/VF is markedly predictive for both ischmic and non-ischemic patients as related to the incidence of post-implantation shock delivery.

## Background

Sudden cardiac death in patients is predominantly caused by ventricular tachycardia (VT)/ventricular fibrillation (VF). Patients who have a low left ventricular ejection fraction (LVEF) and inducible VT during electrophysiologic study (EPS) are at risk of sudden death and may benefit from an implantable cardioverter-defibrillator (ICD) as do patients with low LVEF. However, LVEF's primacy in predicted SCD has been questioned. Recently, cardiac MRI (CMR) has shown that a determination of myocardial core scar via late gadolinium enhancement (LGE) may predict VT/VF with greater precision than LVEF presumably due its ability to define likely sources of macro-rentry by delinieating the 'gray-zone' myocardium.

We hypothesize that LGE depiction of gray-zone scar is more predictive of VT/VF than LGE core scar assessment.

## Methods

A consecutive, retrospective chart review was performed of patients with both a CMR exam for LGE and with post-CMR ICD implantation from 2006-2010 within 30 days. Demographic and clinical events were collected from patient charts and ICD interrogation. Standard LGE (>2SD) and gray-zone (LGE;2-3SD) was manually determined and related as a percent of LV mass to arrhythmic events and ICD therapy.

## Results

A total of 45 subjects met our inclusion criteria. These included patients with both ischemic (n=28) and non-ischemic (n=17) cardiomyopathy. In this population, LVEF was not predictive of ICD therapy in univariate or multivariate analysis (p=NS). In contrast, LGE strongly predicted future ICD therapy (combined anti-tachycardia pacing and defibrillation) in the multivariate logistic model (p=0.02), as well as defibrillation alone (p=0.03). LGE gray zone showed a similar trend for defibrillation but did not reach statistical significance (p=0.06).

## Conclusions

LGE via CMR is markedly predictive for future ICD therapy delivery in patients with non-ischemic and ischemic cardiomyopathy alike. This marker may prove to be an important stratification variable that will greatly enhance current approaches that have traditionally relied soley on LVEF.

## Funding

Internal.

